# REDD1 Silencing Aggravates Aortic Dissection and Promotes VSMC Apoptosis with Autophagy-Related Changes

**DOI:** 10.3390/biomedicines14092061

**Published:** 2026-09-14

**Authors:** Bolai Shen, Xiaoping Xie, Qinyu Chen, Yang Zhou, Jiangxiong Wu, Bowen Li, Zhiwei Wang

**Affiliations:** 1 Department of Cardiovascular Surgery, Renmin Hospital of Wuhan University, Wuhan 430060, China; 2 Laboratory of Cardiovascular Surgery, Renmin Hospital of Wuhan University, 9 Zhangzhidong Road, Wuhan 430060, China

**Keywords:** aortic dissection, REDD1, autophagy, apoptosis, vascular smooth muscle cells

## Abstract

**Background:** Aortic dissection (AD) is a life-threatening vascular disease with high mortality, yet its molecular pathogenesis remains incompletely understood. This study investigated the role of regulated in development and DNA damage responses 1 (REDD1) in vascular smooth muscle cells (VSMCs) apoptosis and the underlying mechanisms. **Methods:** Single-cell RNA sequencing data from GSE222318 were analyzed to investigate the expression levels of REDD1 in VSMCs and its role in inducing apoptosis and autophagy. The target gene was silenced in C57BL/6J mice via tail-vein injection of Adeno-associated virus.β-aminopropionitrile (BAPN) was used for AD induction. Western blotting was used to assess REDD1, LC3B, p62, BAX, BCL-2 in aortic tissues. Aortic histopathological alterations were examined by hematoxylin and eosin (H&E), Elastica van Gieson (EVG), and Masson’s trichrome staining. Immunofluorescence was performed to examine REDD1 expression and its localization in α-SMA-positive vascular smooth muscle cells. REDD1, LC3B, p62, BAX, BCL-2, mTOR, and p-mTOR were also examined by Western blotting in cultured cells. Mitochondrial membrane potential was evaluated by JC-1 staining, and apoptosis was assessed by flow cytometry. **Results:** REDD1 expression levels were significantly increased in human AD tissues and localized predominantly to medial VSMCs. REDD1 levels were positively correlated with BAX and LC3B and negatively correlated with BCL-2 and p62. Knockdown of REDD1 in AD mice was associated with increased levels of BAX and p62 and decreased levels of BCL-2 and LC3B in aortic tissues, accompanied by more severe pathological manifestations. REDD1 silencing in VSMCs increased BAX, p62 and p-mTOR while reducing BCL-2 and LC3B, accompanied by increased apoptosis and loss of mitochondrial membrane potential, whereas rapamycin reduced apoptosis and alleviated mitochondrial injury. **Conclusions:** REDD1 appears to play a protective, compensatory role in AD. Increased REDD1 expression may help preserve mitochondrial membrane potential and attenuate VSMC apoptosis under pathological stress, accompanied by changes in static autophagy-related markers that suggest a possible association with increased autophagic activity. Loss of REDD1 activity promotes mitochondrial injury and VSMC apoptosis, thereby aggravating AD. Targeting REDD1 may therefore represent a potential therapeutic strategy for AD.

## 1. Introduction

Aortic dissection is a relatively insidious but acutely onset vascular disease; once it ruptures, it is extremely difficult to treat successfully [1]. Its major pathological hallmark is degeneration of the aortic media, characterized by elastic fiber fragmentation, extracellular matrix degradation, and loss of vascular smooth muscle cells (VSMCs) [2]. As the predominant cell type in the aortic media, VSMCs maintain extracellular matrix integrity and the mechanical strength of the aortic wall [3]. Excessive VSMC apoptosis weakens aortic wall structure and contributes to the initiation and progression of AD [4]. Although various pathological stimuli can induce VSMC apoptosis, the upstream signaling mechanisms regulating this process remain incompletely elucidated [5].

Autophagy functions as a lysosome-dependent intracellular clearance process that removes damaged organelles and misfolded proteins to maintain cellular homeostasis [6]. In cardiovascular diseases, autophagy is often activated as an adaptive stress response that limits inflammation and apoptosis [7]. Increasing evidence suggests that autophagy is involved in aortic diseases [8]. Experimental studies of aortic dissection (AD) have shown altered autophagic activity, and modulation of autophagy affects vascular smooth muscle cell (VSMCs) survival and aortic wall remodeling, indicating an important role for autophagy in AD pathogenesis [9]. Autophagy and apoptosis are closely interconnected [10]. An intact autophagic level facilitates the clearance of damaged mitochondria and limits the release of pro-apoptotic factors. In contrast, impaired or insufficient autophagy leads to the accumulation of damaged organelles, increased oxidative stress, and enhanced susceptibility to apoptosis [11]. This threshold-dependent shift from autophagy to apoptosis may represent a critical checkpoint determining VSMCs’ fate under pathological conditions, with B-cell lymphoma 2 (BCL-2) family proteins, Beclin-1, and tumor protein p53 (p53) acting as key molecular regulators [12].

Regulated in development and DNA damage responses 1 (REDD1), also known as DNA damage-inducible transcript 4 (DDIT4), is an important stress-responsive protein induced by hypoxia, DNA damage, and metabolic stress [13,14,15]. As a negative regulator of mechanistic target of rapamycin complex 1 (mTORC1), REDD1 promotes autophagy by suppressing mTORC1 activity in tumors and metabolic disorders [16,17]. Loss of REDD1 can lead to aberrant mTORC1 activation, impaired autophagy initiation, and increased susceptibility to apoptotic stimuli [18]. REDD1 also regulates mitochondrial respiratory chain activity and intracellular reactive oxygen species, suggesting a broader role in mitochondrial quality control [19].

However, the role of REDD1 in aortic dissection (AD) remains unknown [18]. Whether REDD1 expression is altered in AD, its predominant cellular source, and its involvement in regulating autophagy and apoptosis have not been fully defined [20]. In this study, we characterized the expression pattern and cellular localization of REDD1 in AD tissues and investigated its role in disease progression by modulating the balance between autophagy and apoptosis. These findings may provide new insights into the pathogenesis of AD and identify REDD1 as a potential therapeutic target.

## 2. Materials and Methods

### 2.1. Bioinformatics Analysis

Single-cell RNA sequencing data from GSE222318 (GEO, https://www.ncbi.nlm.nih.gov/geo/query/acc.cgi?acc=GSE222318, accessed on 7 July 2025) were analyzed using the Seurat R package (version 5.0.0) [21]. The raw gene expression matrix and cell annotation information were imported to construct a Seurat object. After quality control, cells with 800–6000 detected genes and <10% mitochondrial gene content were retained for downstream analysis.

The filtered data were normalized using the LogNormalize method, followed by identification of 2000 highly variable genes, data scaling, and principal component analysis (PCA). Technical variation among samples was mitigated using the Harmony R package (version 1.2.0) [22]. Cell clustering and uniform manifold approximation and projection (UMAP) were then performed using the batch-corrected low-dimensional embeddings. Putative doublets were identified and removed using DoubletFinder, and cell types were annotated according to canonical marker gene expression.

Vascular smooth muscle cells (VSMCs) were further extracted for subpopulation analysis, and key gene expression was compared between the normal and aortic dissection groups. Apoptosis and autophagy activity scores in VSMCs were independently calculated using the AUCell R package (1.24.0). Their distributions between groups were visualized using group-stratified FeaturePlots and violin plots [23,24] (Table S1). Finally, binned correlation analysis was used to evaluate the relationships between key gene expression and the corresponding functional activity scores.

### 2.2. Animals

Approval for all animal experiments was obtained from the Animal Care and Use Committee of Renmin Hospital of Wuhan University (approval no. WDRM-20230904B) and conducted in compliance with the National Institutes of Health Guide for the Care and Use of Laboratory Animals. Three-week-old male C57BL/6J mice were obtained from the Vital River Laboratory Animal Technology Co., Ltd. (Beijing, China) and housed in a specific pathogen-free facility (SPF) under a 12-h light/dark cycle. Mice were monitored four times daily throughout the experimental period, and any deaths were followed by immediate necropsy to determine the cause.

### 2.3. Human Aortic Samples

This study received ethical approval from the Clinical Research Ethics Committee of Renmin Hospital of Wuhan University, Wuhan, China (approval no. WDRY2020-K230), and was conducted in compliance with the 2013 revision of the World Medical Association Declaration of Helsinki. Written informed consent was provided by each patient and donor before enrollment in the study. Aortic specimens were obtained from five patients diagnosed with acute thoracic aortic dissection (AD) who received aortic replacement surgery from December 2020 to December 2021. AD was diagnosed by aortic computed tomography angiography. Patients with connective tissue disorders, including Marfan syndrome and Ehlers–Danlos syndrome, or a family history of aortic disease were excluded. Five non-dissected aortic samples were obtained from redundant donor aortic tissue excised during heart transplantation. These donor tissues were considered suitable controls because the donors had no documented history of circulatory system disorders or other underlying medical conditions, and no gross structural abnormalities were identified in the sampled aortic tissues at the time of collection. All human aortic specimens were processed immediately after surgical excision at the operative site to minimize pre-analytical variation. The tissues were rinsed with phosphate-buffered saline (PBS), divided into aliquots, and immediately frozen, with all handling procedures performed on ice. The frozen specimens were subsequently transported to the laboratory in liquid nitrogen to maintain a continuous low-temperature environment before further analysis. Both acute AD specimens and non-diseased donor aortic tissues obtained during heart transplantation are inherently difficult to acquire; the available human cohort was limited to five samples per group. The baseline characteristics of the AD and donor groups are summarized in Table 1.

**Table 1:** Baseline characteristics of the aortic dissection and donor groups.

Parameters	AD Group (n = 5)	Donor Group (n = 5)	*p* Value
Age (years)	51.4 ± 6.3	48.0 ± 3.2	0.326
Male, n (%)	4 (80.0)	1 (20.0)	0.206
BMI (kg/m^2^)	27.1 ± 3.0	21.3 ± 1.1	0.010 **
Systolic BP (mmHg)	155.4 ± 5.1	116.6 ± 9.5	<0.001 ***
Hypertension, n (%)	5 (100.0)	0 (0.0)	0.008 **
Diabetes, n (%)	0 (0.0)	0 (0.0)	1.000
Smoking history, n (%)	3 (60.0)	0 (0.0)	0.167
Drinking history, n (%)	3 (60.0)	0 (0.0)	0.167
LVEF (%)	58.0 ± 2.7	59.2 ± 1.3	0.412
Hb (g/L)	109.2 ± 15.8	136.8 ± 18.4	0.035 *

Values are presented as mean ± standard deviation or n (%). *p* values were calculated using two-sided Welch *t*-tests for continuous variables and two-sided Fisher exact tests for categorical variables. * *p* < 0.05, ** *p* < 0.01, *** *p* < 0.001.

### 2.4. Animal Models and In Vivo Intervention

The animal experiments consisted of 2 parts: establishment of the aortic dissection (AD) model and investigation of the role of REDD1 in AD. At the experimental endpoint, surviving mice were euthanized by an overdose of isoflurane (5%, 792632, Sigma-Aldrich, St. Louis, MO, USA), and the aortas were harvested for subsequent analyses. To standardize tissue sampling across animals, anatomically matched segments of the descending aorta was collected from the same predefined locations for histological and immunofluorescence analyses. The remaining aortic tissue was reserved for protein extraction and Western blot analysis.

For AD induction, mice were randomly assigned to the control or β-aminopropionitrile (BAPN) group (n = 15 per group). Mice in the BAPN group were fed a diet containing 0.25% BAPN (Shulaibao Biotechnology, Wuhan, China) for 4 weeks based on previously established BAPN-induced AD models [25,26,27], whereas control mice received a standard diet. The BAPN-containing diet was replaced daily to minimize degradation. Survival was monitored throughout the 28-day modeling period. Animals that died during the observation period underwent immediate necropsy, and their aortas were collected for subsequent assessment. No animals were excluded after group allocation.

To further investigate the role of REDD1 in vascular smooth muscle cells during AD, an adeno-associated viral vector targeting REDD1 (AAV-shREDD1) in the mouse aorta was generated by Corues Biotechnology (Shanghai, China). Mice were randomly assigned to the AAV-shNC or AAV-shREDD1 group (n = 15 per group) and received the corresponding vectors by tail-vein injection (1 × 10^12^ vg per mouse). Three days after viral administration, all mice were fed a diet containing 0.25% BAPN for 4 weeks. Survival was monitored throughout the 28-day experimental period. Animals that died during follow-up underwent immediate necropsy, and their aortas were retained for subsequent analyses. No animals were excluded after group allocation.

Animals were considered eligible for exclusion only in the event of severe procedure-related complications unrelated to AD, accidental death unrelated to the experimental intervention, or loss or severe damage of aortic tissue that precluded subsequent assessment. For the AAV intervention experiment, unsuccessful tail-vein injection was additionally considered an exclusion criterion. Deaths associated with AD or aortic rupture during the observation period were not considered exclusion events and were retained in the survival and AD incidence analyses. No animals met these exclusion criteria, and no animals were excluded after randomization. Animal allocation and disease outcomes are summarized in Table 2.

**Table 2:** Animal allocation and disease outcomes.

Group	Initial n	Deaths	Survived to Endpoint	TAD Cases	Non-TAD Cases	TAD Incidence
NC	15	0	15	0	15	0%
BAPN	15	5	10	9	6	60.0%
AAV-shNC	15	4	11	8	7	53.3%
AAV-shREDD1	15	12	3	12	3	80.0%

Notes: All animals allocated to each group were included in the survival and TAD incidence analyses. Aortic tissues collected from both surviving animals at the experimental endpoint and animals that died during the observation period were retained for Western blot analysis. For Western blotting, aortic tissues from five mice within the same group were pooled to generate one independent protein sample, resulting in three pooled samples per group. Both TAD and non-TAD animals were included in the Western blot analysis. TAD and non-TAD aortic samples were appropriately distributed among the pooled samples to ensure a representative composition within each experimental group.

### 2.5. Mouse Aortic Smooth Muscle Cell Culture

Mouse aortic smooth muscle cells (MOVAS) (CRL-2797, ATCC, Manassas, VA, USA) were cultured in Dulbecco’s modified Eagle’s medium (DMEM) (6124322, Gibco Life Technologies, Grand Island, NY, USA) containing 10% fetal bovine serum (FBS) (BS1612-109, BIOEXPLORER, Caesarea, Israel) and 1% 100× penicillin–streptomycin solution (G4003-100ML, Servicebio, Wuhan, China) at 37 °C in a humidified atmosphere containing 5% CO_2_. MOVAS were treated with 6μM angiotensin II (Ang II) or 100 nM rapamycin (RAPA) for 24 h. Cells in the vehicle control group received 0.01% dimethyl sulfoxide (DMSO) (GC203006-10ml, Servicebio, China).

### 2.6. RNA Interference and Transfection

REDD1-targeting and negative control short hairpin RNA (shRNA) plasmids were synthesized by Corues Biotechnology (Shanghai, China). MOVAS were seeded in six-well plates at approximately 1.50 × 10^6^ cells per well and transfected with the corresponding shRNA plasmids using Lipofectamine 3000 (L3000001, Invitrogen, Carlsbad, CA, USA) according to the manufacturer’s instructions. Before transfection, cells were washed with phosphate-buffered saline (PBS). Lipofectamine 3000 and shRNA plasmids were separately diluted in Opti-MEM reduced-serum medium (31985070, Gibco Life Technologies, Grand Island, NY, USA), mixed, and added to the cells at a final shRNA concentration of 10 nM. After 9 h of transfection, the transfection medium was replaced with complete culture medium. Cells were then maintained in complete medium until they reached approximately 50% confluence. Subsequently, cells were treated with either 6 μM Ang II alone or 6 μM Ang II together with 100 nM rapamycin for 24 h before being harvested for downstream analyses. Cells were subsequently harvested, and transfection efficiency was assessed by Western blotting. The REDD1-targeting shRNA sequence was as follows: shRNA1 5′-TCAGAGTCATCAAGAAGAAAC-3′; shRNA2 5′-TCTTCCTTGGTCCCTGGTTAC-3′; shRNA3 5′-TCCGAGCAGCTGCTCATTGAA-3′.

### 2.7. Western Blotting

For Western blot analysis of mouse aortic tissues, aortic tissues from five mice within the same experimental group were pooled before protein extraction to obtain sufficient total protein for the assessment of all target proteins included in the study. Animals were not excluded from the biochemical analyses on the basis of whether TAD was confirmed at the experimental endpoint; therefore, aortic tissues from both TAD and non-TAD animals were retained for Western blot analysis. Thus, each group generated three independent pooled aortic samples, with each pooled sample comprising tissues from five mice. Each pooled sample was processed independently throughout protein extraction, electrophoresis, immunoblotting, and densitometric analysis. Cell and aortic tissue samples were lysed using radioimmunoprecipitation assay (RIPA) buffer supplemented with phosphatase inhibitor (G2007-1ML, Servicebio, China), phenylmethylsulfonyl fluoride (PMSF) (G2008-1ML, Servicebio, China), and Protease Inhibitor Cocktail (MedChemExpress, HY-K0010, Wuhan, China). Lysates were centrifuged at 12,000 rpm for 15 min at 4 °C, and protein concentrations in the supernatants were determined using a bicinchoninic acid (BCA) assay kit (P0010, Beyotime, Shanghai, China). Total proteins were mixed with sodium dodecyl sulfate (SDS) sample loading buffer (G2075-1ML, Servicebio, China) and heated at 95 °C for 10 min. Equal amounts of protein were separated by 6–12% sodium dodecyl sulfate–polyacrylamide gel electrophoresis (SDS–PAGE) and transferred onto polyvinylidene difluoride (PVDF) (Millipore, IPVH00010, Burlington, MA, USA) membranes at room temperature. The membranes were incubated with the indicated primary antibodies at 4 °C with gentle agitation for 12–16 h. Primary antibodies used in this study included: anti-regulated in development and DNA damage responses 1 (REDD1/DDIT4) (1:7000, 10638-1-AP, Proteintech, Wuhan, China), anti-BCL2-associated X protein (BAX) (1:60,000, 50599-2-Ig, Proteintech, China), anti-B-cell lymphoma 2 (BCL-2) (1:2000, 26593-1-AP, Proteintech, China), anti-sequestosome 1 (SQSTM1/p62) (1:30,000, 66184-1-Ig, Proteintech, China), anti-microtubule-associated protein 1 light chain 3 beta (LC3B) (1:2000, 18725-1-AP, Proteintech, China), anti-mechanistic target of rapamycin (mTOR) (1:9000, 28273-1-AP, Proteintech, China), anti-phospho-mechanistic target of rapamycin (p-mTOR) (1:3000, 28881-1-AP, Proteintech, China), anti-glyceraldehyde-3-phosphate dehydrogenase (GAPDH) (1:23,000, 10494-1-AP, Proteintech, China), and anti-beta-actin (ACTB) (1:7000, 20536-1-AP, Proteintech, China). After incubation with the primary antibodies, the membranes were treated with the corresponding HRP-conjugated goat anti-rabbit IgG (1:25,000, RGAR001, Proteintech, China) or HRP-conjugated goat anti-mouse IgG (1:25,000, RGAM001, Proteintech, China) for 1 h at room temperature, according to the host species of the primary antibodies. Protein bands were visualized using an ultrasensitive enhanced chemiluminescence (ECL) reagent (BL523B, Biosharp, Beijing, China). Western blot band intensities were quantified using ImageJ software (version 1.53; National Institutes of Health, Bethesda, MD, USA). Background-corrected densitometric values of target proteins were normalized to the corresponding internal loading control, GAPDH or β-actin, as indicated in each experiment. The LC3B-II/LC3B-I ratio was calculated from the densitometric intensities of the corresponding bands, whereas phosphorylated mTOR was normalized to total mTOR for assessment of mTOR phosphorylation. Quantitative values were obtained from independently processed biological replicates and used for statistical analysis.

### 2.8. Histopathology

To minimize anatomical sampling variability, aortic segments were consistently collected from the same predefined location of the descending aorta in all animals, fixed in 4% paraformaldehyde fixation solution (PFA) (G1101-500ML, Servicebio, China) for 24 h at room temperature, embedded in paraffin, and transversely sectioned at a thickness of 2 μm. After deparaffinization and rehydration, the sections were stained with hematoxylin and eosin (H&E), Elastica van Gieson (EVG), and Masson’s trichrome to evaluate histopathological alterations.

### 2.9. Immunofluorescence

After deparaffinization and rehydration, aortic sections were permeabilized with 0.5% Triton X-100 and blocked with blocking solution (G2052-500ML, Servicebio, China). Sections were incubated with primary antibodies against REDD1 (1:500, 10638-1-AP, Proteintech, China) and α-smooth muscle actin (α-SMA) (1:500, 67735-1-Ig, Proteintech, China) at 4 °C for 12–16 h, followed by species-appropriate fluorophore-conjugated secondary antibodies for 1–2 h at room temperature. The sections were then mounted with antifade mounting medium containing 4′,6-diamidino-2-phenylindole (DAPI), and fluorescence signals were visualized and recorded using a fluorescence microscope (BX53, Olympus Corporation, Hachioji, Tokyo, Japan).

### 2.10. Mitochondrial Membrane Potential Assay

Mitochondrial membrane potential (ΔΨm) was assessed using a JC-1 (5,5′,6,6′-Tetrachloro-1,1′,3,3′-tetraethylbenzimidazolylcarbocyanine iodide) Mitochondrial Membrane Potential Assay Kit (C1475S, Beyotime, China). The cell samples were exposed to JC-1 staining solution at 37 °C for 20 minutes, and fluorescence signals were visualized by fluorescence microscopy. In cells with intact ΔΨm, JC-1 accumulated in mitochondria and formed aggregates emitting red fluorescence, whereas mitochondrial depolarization caused JC-1 to remain as monomers emitting green fluorescence.

### 2.11. Flow-Cytometric Analysis of Apoptosis

Cell apoptosis was evaluated using Annexin V–FITC (Fluorescein isothiocyanate) /propidium iodide (PI) double staining using a Annexin V-FITC Apoptosis Detection Kit (C1062, Beyotime, China). Upon completion of the designated treatments, cells were collected and rinsed twice with ice-cold PBS and resuspended in 1× binding buffer at 1 × 10^6^ cells/mL. A 100-μL aliquot containing approximately 1 × 10^5^ cells was collected into a flow cytometry tube and subsequently incubated with 5 μL Annexin V–FITC and 5 μL PI. Samples were gently mixed and incubated for 15 min at 25 °C in the dark. Subsequently, 400 μL of 1× binding buffer was added, and the samples were analyzed by flow cytometry (FongCytec2080, Challen Biotechnology, Beijing, China) within 1 h. For data analysis, cellular debris was excluded on the basis of forward-scatter and side-scatter characteristics, and the main cell population was subsequently analyzed using Annexin V-FITC versus PI fluorescence plots. Cells were classified into four populations according to Annexin V-FITC and PI staining: Annexin V-FITC^−^/PI^−^ cells were considered viable cells, Annexin V-FITC^+^/PI^−^ cells were considered early apoptotic cells, Annexin V-FITC^+^/PI^+^ cells were considered late apoptotic or secondary necrotic cells, and Annexin V-FITC^−^/PI^+^ cells were considered primarily necrotic cells. The total apoptotic fraction was calculated as the sum of the early and late apoptotic cell populations. The resulting apoptotic percentages were exported and subsequently analyzed and plotted using GraphPad Prism 10.0.

### 2.12. Statistical Analysis

Quantitative measurements are reported as mean ± standard error of the mean (SEM), with a minimum of three biologically independent replicates included in each experiment. The distributional characteristics of the data were first examined by the Shapiro–Wilk test. For two-group comparisons, an unpaired Student’s *t*-test was selected when the data conformed to a normal distribution; otherwise, significance was determined using the Mann–Whitney U test. For comparisons among three or more groups, data satisfying the assumptions of parametric testing were analyzed using one-way analysis of variance (ANOVA), followed by Tukey’s post hoc test for multiple comparisons. Data failing to meet these assumptions were instead examined by the Kruskal–Wallis test, followed by Dunn’s multiple-comparisons test. Survival outcomes were compared using the log-rank test. Statistical significance was set at *p* < 0.05. Calculations and statistical testing were carried out in GraphPad Prism 10.0.

## 3. Results

### 3.1. REDD1 Is Upregulated in VSMCs from Aortic Dissection and Is Associated with Apoptosis and Autophagy States

**Figure 1:**
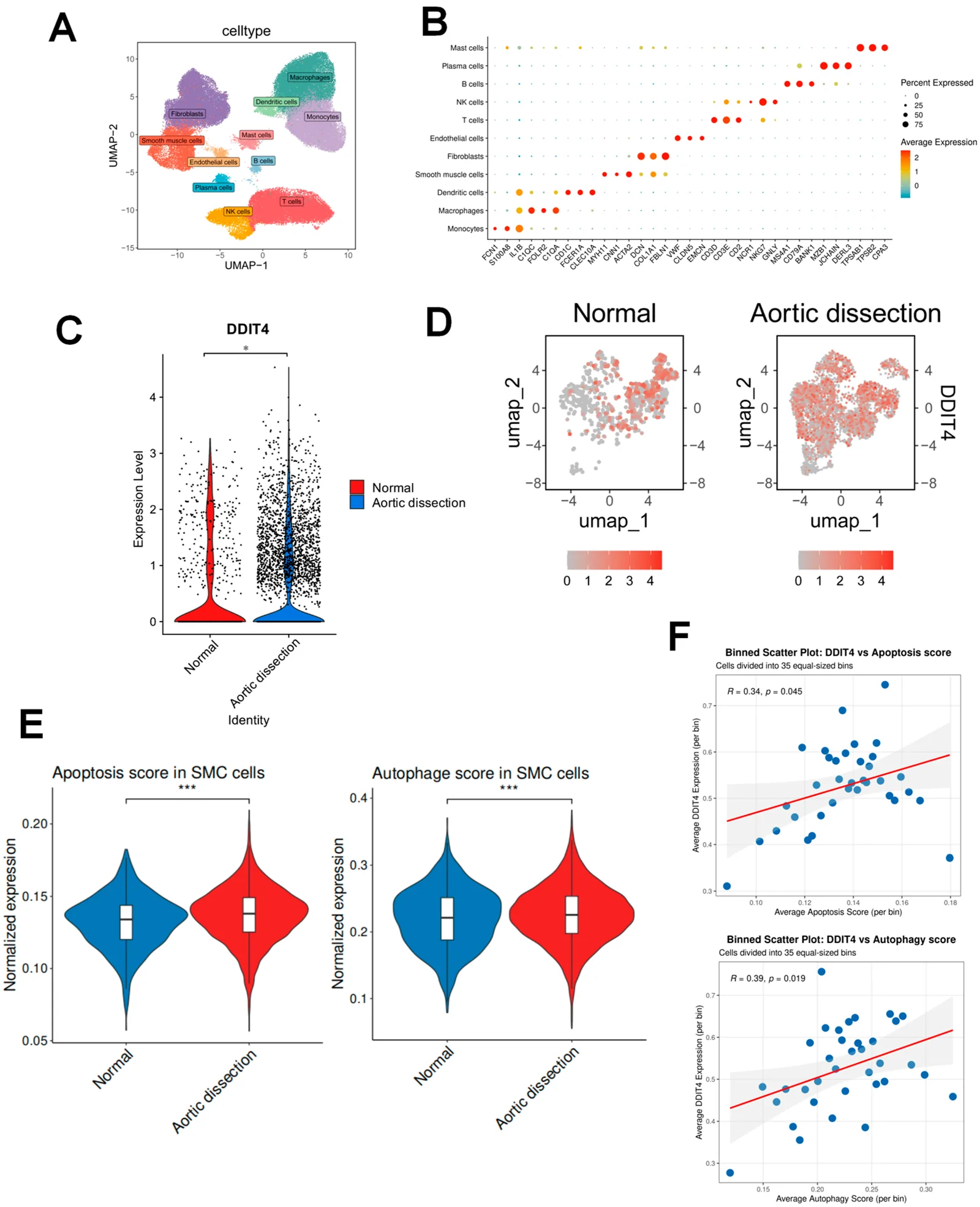
REDD1 is upregulated in VSMCs from AD and is associated with apoptosis and autophagy states. (**A**) UMAP plot of GSE222318 displaying the cellular composition of aortic tissues in GSE222318. (**B**) Dot plot illustrating the expression profiles of canonical marker genes across the identified cell populations. (**C**) Violin plot showing the expression levels of REDD1 (DDIT4) in VSMCs between Normal and Aortic dissection groups. (**D**) UMAP plots depicting the expression distribution of REDD1 (DDIT4) in VSMCs. (**E**) Violin plots comparing the Apoptosis score and Autophagy score in VSMCs between the Normal and Aortic dissection groups. (**F**) Binned scatter plots showing the correlation between REDD1 (DDIT4) expression and Apoptosis/Autophagy scores in VSMCs from the disease group. Each dot represents an equal-number cell bin; red lines indicate linear regression fits. Group differences displayed in the violin plots were examined with the Wilcoxon rank-sum test, whereas associations shown in the scatter plots were quantified using Pearson’s correlation. * *p* < 0.05, *** *p* < 0.001.

To characterize the cellular distribution of REDD1 and explore its potential functional relevance in aortic dissection, single-cell RNA sequencing data deposited in GEO under accession GSE222318 were subjected to downstream analysis. Cells were annotated as monocytes, macrophages, dendritic cells, VSMCs, fibroblasts, endothelial cells, T cells, natural killer cells, B cells, plasma cells, and mast cells (Figure 1A,B). Examination of the VSMC compartment revealed a higher distribution of REDD1 transcript expression in cells derived from aortic dissection samples than in those from normal controls (Figure 1C). Grouped FeaturePlots also showed a broader distribution of REDD1-positive VSMCs in aortic dissection (Figure 1D).

Both apoptosis and autophagy scores were significantly increased in VSMCs from the aortic dissection group (Figure 1E). In diseased VSMCs, REDD1 expression was positively correlated with the apoptosis score (R = 0.34, *p* = 0.045) and autophagy score (R = 0.39, *p* = 0.019) (Figure 1F). These findings suggest that REDD1 is associated with apoptosis- and autophagy-related functional states of VSMCs in aortic dissection.

### 3.2. REDD1 Is Upregulated in Human Dissected Aortas and Is Associated with Autophagy- and Apoptosis-Related Changes

**Figure 2:**
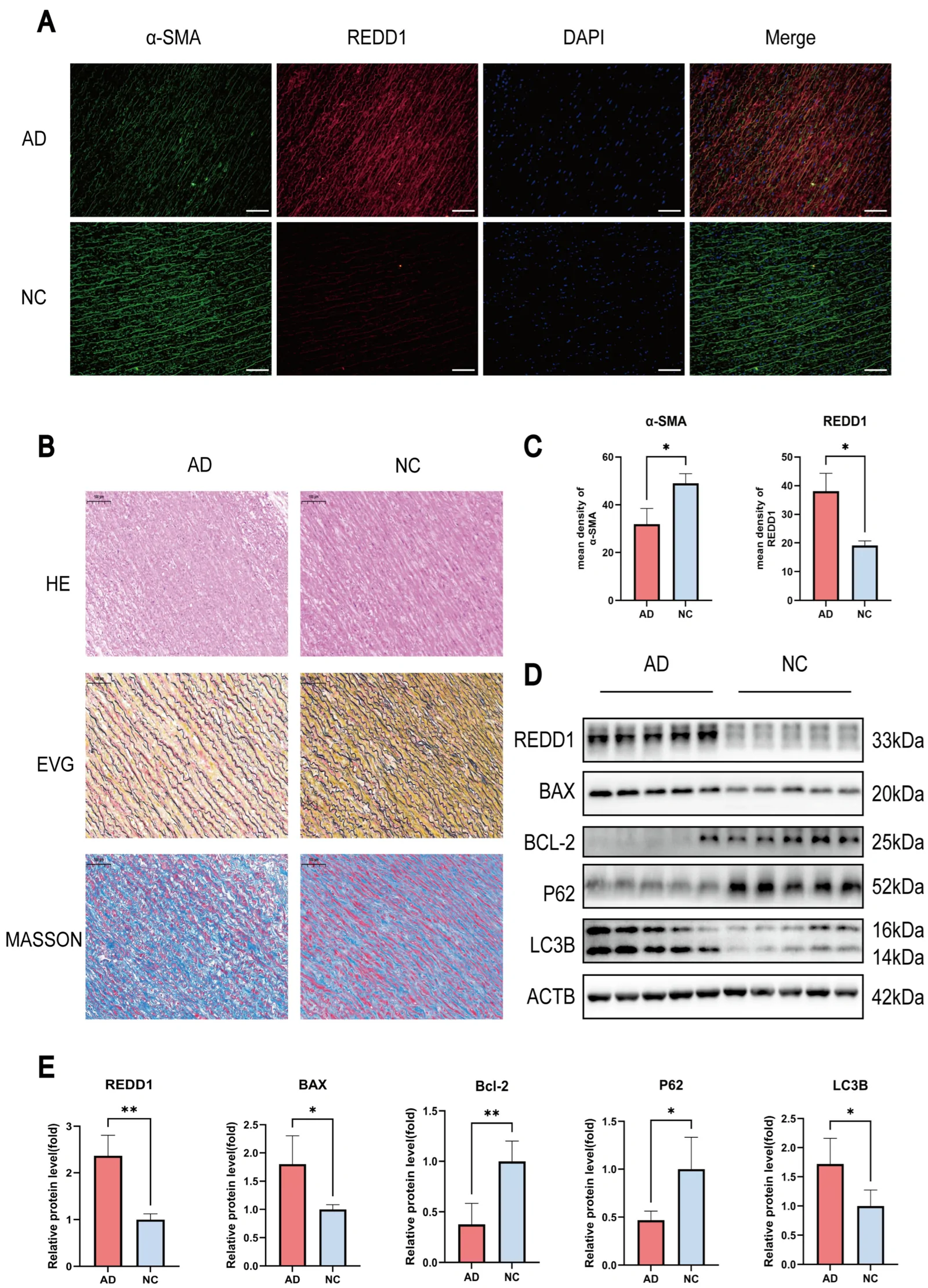
REDD1 is upregulated in human dissected aortas and is associated with autophagy- and apoptosis-related changes. (**A**) Representative immunofluorescence images of α-SMA and REDD1 in AD and NC aortic tissues. Nuclei were stained with DAPI. Magnification: 20×; scale bar: 100 µm. (**B**) Representative histological images of HE, EVG, and Masson’s trichrome staining of aortic tissues from AD and NC groups (Magnification: 20×; scale bar: 100 µm). (**C**) Quantitative analysis of the mean fluorescence density of α-SMA and REDD1 from the immunofluorescence experiments shown in (**A**). (**D**) Western blot analysis of REDD1, BAX, BCL-2, p62, and LC3B protein levels in AD and NC aortic tissues. (**E**) Quantification of the relative protein levels of REDD1, BAX, BCL-2, p62, and LC3B from the Western blots shown in (**D**). Values represent mean ± SEM; statistical significance was determined using an unpaired Student’s *t*-test. * *p* < 0.05, ** *p* < 0.01 vs. NC group (n = 5 per group).

Bioinformatics analysis identified REDD1 as a candidate key molecule, and its expression was further validated in aortic tissues from patients with aortic dissection (AD). Immunofluorescence staining showed markedly increased REDD1 expression in the aortic media of patients with AD, whereas α-smooth muscle actin (α-SMA), a marker of vascular smooth muscle cells (VSMCs), was reduced compared with controls. The immunofluorescence images further revealed that REDD1 signals were mainly distributed within α-SMA-positive regions of the aortic media (Figure 2A,C). Histological examination showed marked structural disruption of the aortic media, while EVG staining revealed pronounced fragmentation and disorganization of elastic fibers in AD samples. Masson’s trichrome staining showed extensive collagen deposition accompanied by severe elastic fiber loss (Figure 2B). Consistent with the immunofluorescence results, Western blotting confirmed increased REDD1 protein levels in AD tissues. BCL-2-associated X protein (BAX) was upregulated, whereas BCL-2 was downregulated, indicating enhanced apoptosis. In parallel, the microtubule-associated protein 1 light chain 3 beta-II/I (LC3B-II/I) ratio was increased and p62 (Sequestosome 1) expression was reduced, indicating corresponding changes in static autophagy-related markers under pathological conditions (Figure 2D,E).

### 3.3. REDD1 Is Upregulated in the Aorta of BAPN-Treated Mice During Aortic Dissection Progression

**Figure 3:**
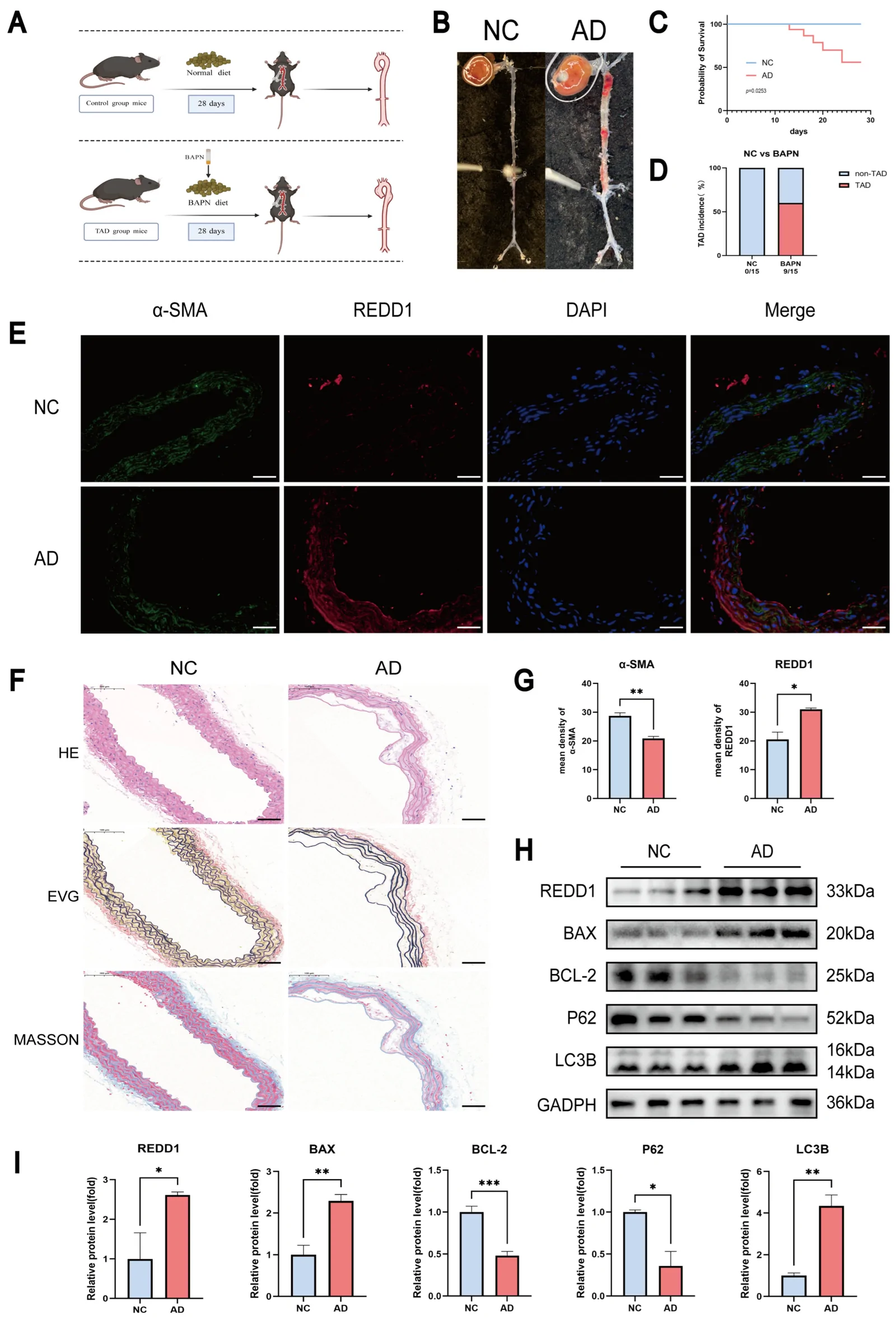
REDD1 is upregulated in the aorta of BAPN-treated mice during aortic dissection progression. (**A**) Schematic illustration of the experimental protocol for establishing the BAPN-induced TAD mouse model. (**B**) Representative gross morphological images of aortas from the NC and AD groups. (**C**) Kaplan–Meier survival curves of mice in the NC and AD groups during the 28-day observation period. (**D**) Incidence of TAD in NC and AD mouse models. (**E**) Representative immunofluorescence images showing the expression and co-localization of α-SMA and REDD1 in aortic tissues. Nuclei were counterstained with DAPI. Magnification: 20×; scale bar: 100 µm. (**F**) Representative histological images of HE, EVG, and Masson’s trichrome staining of aortic tissues from NC and AD groups. Magnification: 20×; scale bar: 100 µm. (**G**) Quantitative analysis of the mean fluorescence density of α-SMA and REDD1 based on the immunofluorescence results shown in (**E**). (**H**) Western blot analysis of REDD1, BAX, BCL-2, p62, and LC3B protein levels in aortic tissues from NC and AD groups. (**I**) Quantification of the relative protein levels of REDD1, BAX, BCL-2, p62, and LC3B from the Western blots shown in (**H**). Data are presented as mean ± SEM; statistical analysis was performed using an unpaired Student’s *t*-test. * *p* < 0.05, ** *p* < 0.01, *** *p* < 0.001 vs. NC group (n = 3 per group).

To validate the association between REDD1 and aortic dissection (AD) progression in vivo, a BAPN-induced AD model was established in C57BL/6J mice by dietary BAPN administration (Figure 3A). Gross examination showed typical double-lumen formation and intramural hematoma in the thoracic aortas of BAPN-treated mice (Figure 3B). Survival was significantly reduced in the BAPN group, with a cumulative AD incidence exceeding 50% (Figure 3C,D). Histological analysis revealed marked aortic wall disorganization and loss of medial components in BAPN-treated mice. Elastica van Gieson (EVG) staining showed disordered elastic fiber architecture and increased fragmentation, whereas Masson’s trichrome staining demonstrated increased collagen deposition accompanied by severe elastic fiber degradation (Figure 3F). Immunofluorescence staining showed increased REDD1 expression and reduced α-smooth muscle actin (α-SMA) in the aortic media. REDD1 colocalized with α-SMA-positive medial vascular smooth muscle cells (VSMCs), consistent with the findings in human AD tissues (Figure 3E,G). Western blotting further confirmed increased REDD1 protein expression in BAPN-treated aortas. BAX was upregulated, whereas BCL-2 was downregulated. In parallel, the LC3B-II/LC3B-I ratio was increased and p62 expression was reduced (Figure 3H,I), suggesting increased levels of static autophagy-related markers accompanied by activation of apoptotic signaling. Collectively, these findings support the involvement of REDD1 in the pathological progression of AD in vivo.

### 3.4. REDD1 Silencing Aggravates BAPN-Induced Aortic Dissection in Mice

**Figure 4:**
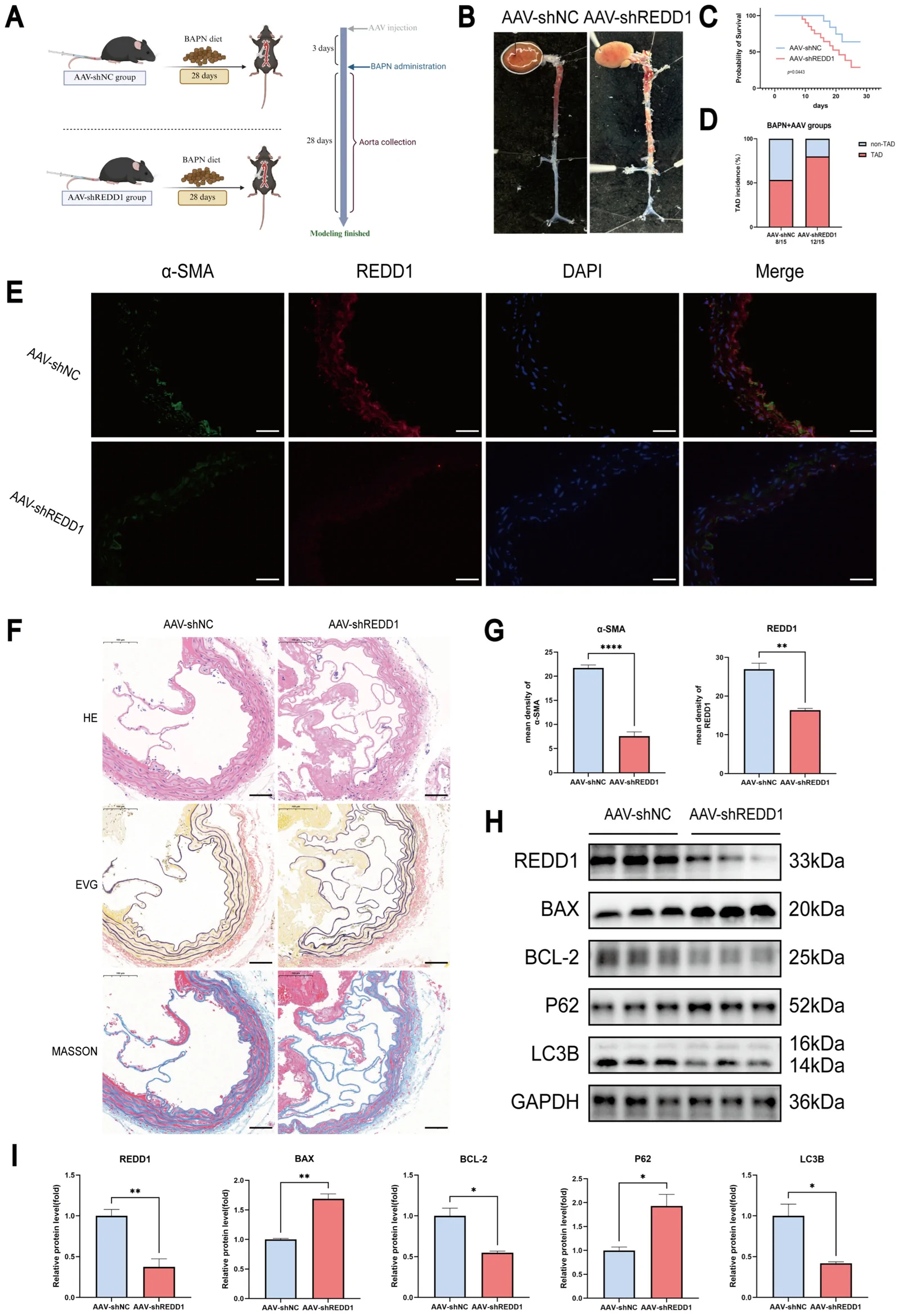
REDD1 silencing aggravates BAPN-induced aortic dissection in mice. (**A**) Schematic illustration of the experimental timeline. Mice were injected with AAV-shNC or AAV-shREDD1 via the tail vein, followed by BAPN administration for 28 days to establish the aortic dissection model. (**B**) Representative gross morphological images of aortas from the AAV-shNC and AAV-shREDD1 groups. (**C**) Kaplan–Meier survival curves of mice in the AAV-shNC and AAV-shREDD1 groups during the 28-day observation period. (**D**) Incidence of TAD in the AAV-shNC and AAV-shREDD1 groups. (**E**) Representative immunofluorescence images of α-SMA and REDD1 in aortic tissues. Nuclei were counterstained with DAPI. Magnification: 20×; scale bar: 100 µm. (**F**) Representative histological images of HE, EVG, and Masson’s trichrome staining of aortic tissues. Magnification: 20×; scale bar: 100 µm. (**G**) Quantitative analysis of the mean fluorescence density of α-SMA and REDD1 from the immunofluorescence results shown in (**E**). (**H**) Western blot analysis of REDD1, BAX, BCL-2, p62, and LC3B protein levels in aortic tissues. (**I**) Quantification of the relative protein levels of REDD1, BAX, BCL-2, p62, and LC3B from the Western blots shown in (**H**). Data are presented as mean ± SEM. Statistical significance for two-group comparisons was determined using an unpaired Student’s *t*-test. * *p* < 0.05, ** *p* < 0.01, **** *p* < 0.0001 vs. AAV-shNC group (n = 3 per group).

To investigate the role of REDD1 in aortic dissection (AD) progression, C57 mice were transduced with recombinant adeno-associated virus AAV-shREDD1 and subsequently fed BAPN to establish the AD model (Figure 4A). Gross examination showed more pronounced aortic dilatation and remodeling, broader aortic involvement, and more severe intramural hematoma formation in the AAV-shREDD1 group than in controls (Figure 4B). Survival analysis further showed an earlier onset of death, a markedly shortened median survival time, and a significantly higher cumulative incidence of AD in the AAV-shREDD1 group (Figure 4C,D). Immunofluorescence staining showed markedly reduced REDD1 fluorescence in the aortic media of the AAV-shREDD1 group, accompanied by decreased expression of the smooth muscle cell marker α-SMA, indicating extensive loss of medial vascular smooth muscle cells (VSMCs) (Figure 4E,G). Histological analysis by hematoxylin and eosin (H&E) and Elastica van Gieson (EVG) staining further revealed aggravated disorganization and disruption of the aortic wall after REDD1 knockdown. Masson’s trichrome staining showed more severe elastic fiber degradation in the AAV-shREDD1 group than in controls (Figure 4F). Together, these pathological changes indicate that REDD1 silencing markedly compromises aortic wall integrity. Western blotting further showed that REDD1 silencing resulted in a static autophagy-related marker profile indicative of reduced autophagy, characterized by a decreased LC3B-II/LC3B-I ratio and increased p62 accumulation in aortic tissues. Meanwhile, REDD1 knockdown altered the mitochondrial apoptotic pathway, with increased BAX expression, decreased BCL-2 expression. These findings suggest that defective autophagy initiation following REDD1 silencing may be mechanistically linked to enhanced apoptotic signaling (Figure 4H,I).

### 3.5. REDD1 Silencing Promotes VSMC Apoptosis, Accompanied by Reduced Mitochondrial Membrane Potential and Altered Autophagic Markers

**Figure 5:**
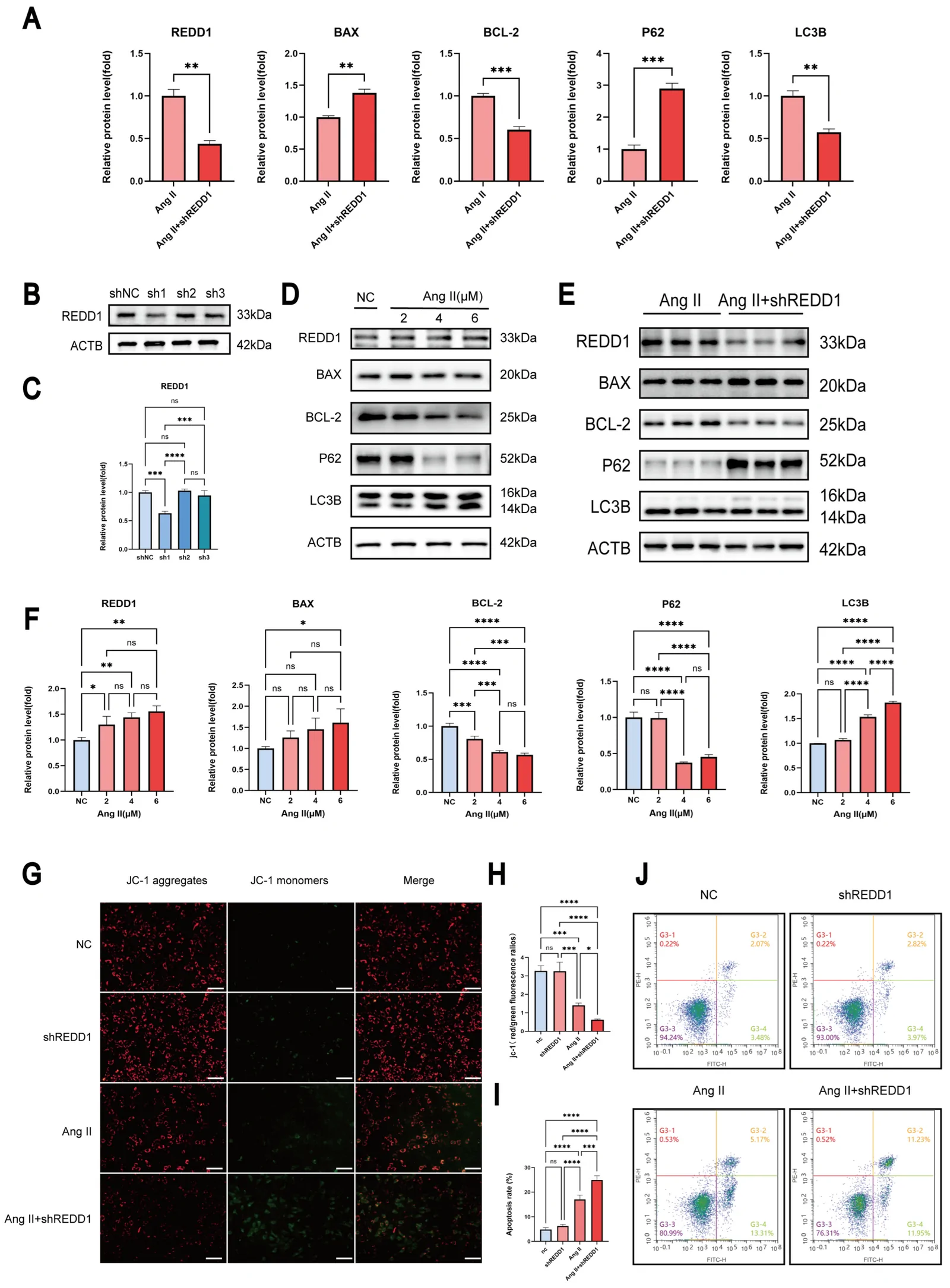
REDD1 silencing promotes VSMC apoptosis, accompanied by reduced mitochondrial membrane potential and altered autophagic markers. (**A**) Quantification of REDD1, BAX, BCL-2, p62, and LC3B protein levels in VSMCs treated with Ang II or Ang II + shREDD1 (corresponding to (**E**)). (**B**) Western blot validation of REDD1 knockdown efficiency using three shRNAs (sh1, sh2, sh3). (**C**) Quantification of REDD1 protein levels corresponding to the Western blots in (**B**). (**D**) Protein expression of REDD1, BAX, BCL-2, p62, and LC3B in VSMCs treated with increasing Ang II concentrations (0–6 µM). (**E**) Representative Western blots of the indicated proteins in VSMCs under Ang II and Ang II + shREDD1 conditions. (**F**) Quantitative analysis of protein levels from the experiments shown in (**D**). (**G**) JC-1 staining of VSMCs across four groups (NC, shREDD1, Ang II, Ang II + shREDD1). Red: Aggregates (intact mitochondrial membrane potential); Green: Monomers (depolarization). Magnification: 40×; scale bar: 50 µm. (**H**) Quantitative analysis of the JC-1 aggregates/monomers ratio in VSMCs. (**I**) Quantitative analysis of apoptosis rates in VSMCs (corresponding to (**J**)). (**J**) Representative flow cytometry plots assessing apoptosis via Annexin V-FITC/PI staining. Data are presented as mean ± SEM. Statistical significance for two-group comparisons was determined using an unpaired Student’s *t*-test; for multiple group comparisons, one-way ANOVA followed by post hoc tests was used. * *p* < 0.05, ** *p* < 0.01, *** *p* < 0.001, **** *p* < 0.0001; ns indicates no significant difference.

Given the marked increase in REDD1 expression in medial VSMCs within AD lesions, we further examined its functional role in vitro. Three REDD1-targeting shRNAs were designed and screened, and the shRNA with the highest knockdown efficiency, as confirmed by Western blotting, was selected for subsequent experiments (Figure 5B,C). To determine the optimal condition for apoptosis induction, VSMCs were treated with increasing concentrations of Ang II (0–6 μM). Apoptotic signaling was most pronounced at 6 μM Ang II (Figure 5D,F). Under this condition, REDD1 knockdown combined with Ang II treatment reduced the LC3B-II/LC3B-I ratio and increased p62 accumulation compared with Ang II treatment alone, indicating reduced levels of static autophagy-related markers consistent with reduced autophagy. Meanwhile, BAX was upregulated and BCL-2 was downregulated, indicating enhanced apoptotic signaling (Figure 5A,E). These changes were consistent with the preceding in vivo findings. JC-1 staining further showed that REDD1 knockdown significantly reduced the mitochondrial membrane potential (ΔΨm) in VSMCs, indicating impaired mitochondrial function (Figure 5G,H). Flow cytometry confirmed a marked increase in the proportion of apoptotic cells following REDD1 silencing (Figure 5I,J). Together, these results indicate that REDD1 knockdown is associated with a static autophagy-related marker profile suggestive of reduced autophagic activity in VSMCs and is accompanied by aggravated mitochondrial injury and increased apoptosis.

### 3.6. Rapamycin Treatment Attenuates REDD1 Knockdown-Induced VSMC Apoptosis and Partially Restores Mitochondrial Membrane Potential

**Figure 6:**
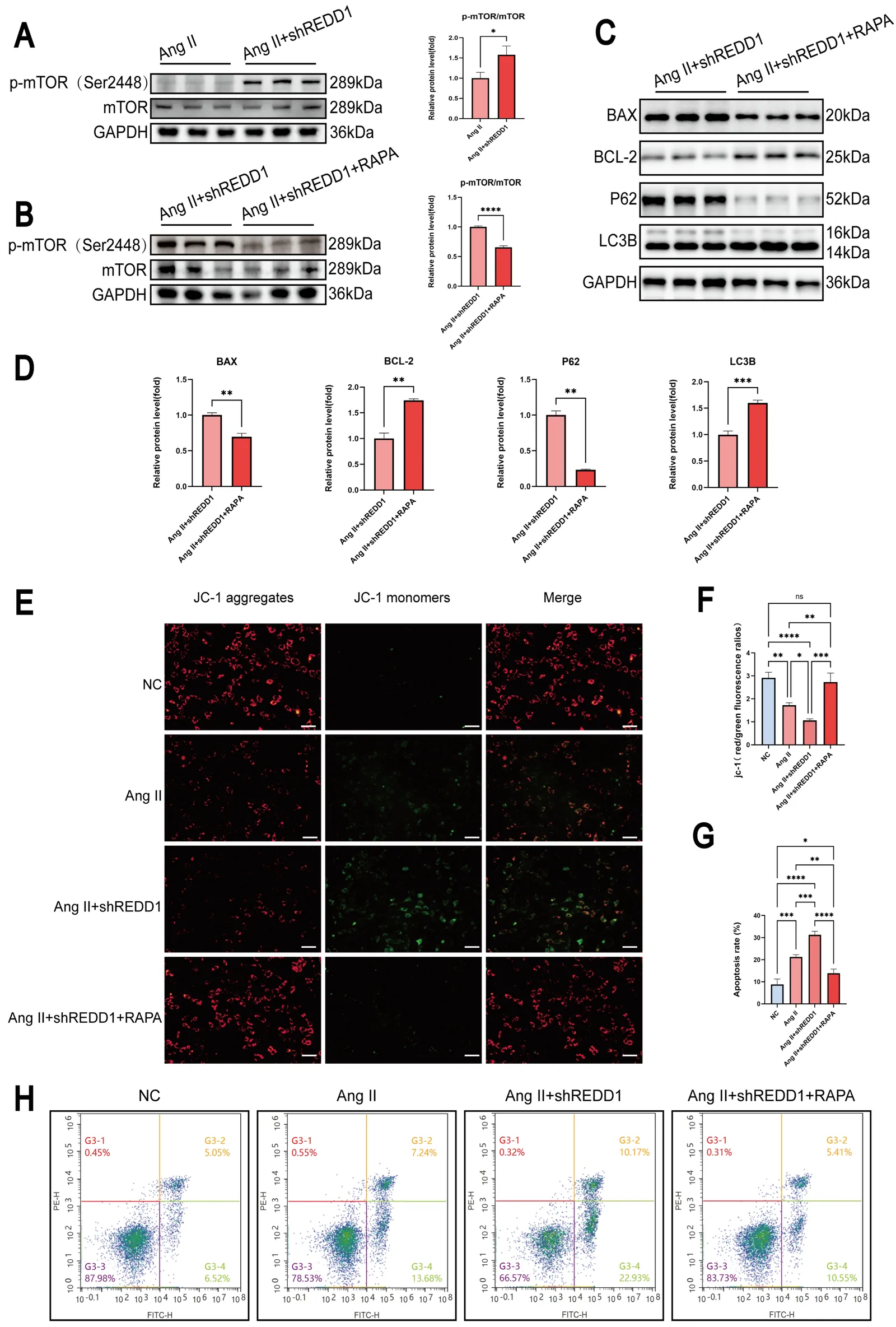
Rapamycin treatment attenuates REDD1 knockdown-induced VSMC apoptosis and partially restores mitochondrial membrane potential. (**A**) Western blot analysis of p-mTOR and total mTOR protein levels in VSMCs under Ang II, Ang II + shREDD1 conditions. GAPDH served as an internal control. (**B**) Western blot analysis of p-mTOR and total mTOR protein levels in VSMCs under Ang II + shREDD1, Ang II + shREDD1 + RAPA conditions. GAPDH served as an internal control. (**C**) Western blot analysis of BAX, BCL-2, p62, and LC3B protein levels in VSMCs treated with Ang II + shREDD1 or Ang II + shREDD1 + RAPA. (**D**) Quantification of the relative protein levels of BAX, BCL-2, p62, and LC3B from the Western blots shown in (**C**). (**E**) Representative fluorescence images of JC-1 staining in VSMCs across the indicated groups. Red: JC-1 aggregates (intact mitochondrial membrane potential); Green: JC-1 monomers (depolarization). Magnification: 40×; Scale bar: 50 µm. (**F**) Quantitative analysis of the JC-1 aggregates/monomers fluorescence intensity ratio. (**G**) Quantitative analysis of apoptosis rates in VSMCs from the flow cytometry results shown in (**H**). (**H**) Representative flow cytometry plots assessing apoptosis via Annexin V-FITC/PI staining. Data are presented as mean ± SEM. Statistical significance for two-group comparisons was determined using an unpaired Student’s *t*-test; for multiple group comparisons, one-way ANOVA followed by post hoc tests was used. * *p* < 0.05, ** *p* < 0.01, *** *p* < 0.001, **** *p* < 0.0001; ns indicates no significant difference.

REDD1 is a negative regulator of mTOR signaling, whereas mTOR activation suppresses autophagy initiation [28]. To determine whether the effects of REDD1 loss were mediated through this pathway, cells were treated with the mTOR inhibitor rapamycin [29]. Under Ang II stimulation, REDD1 knockdown increased mTOR phosphorylation at Ser2448, indicating enhanced mTOR signaling, whereas rapamycin prevented this increase (Figure 6A,B). Rapamycin also partially restored autophagy-related markers, with increased LC3B-II and reduced p62, and attenuated apoptotic signaling by decreasing the BAX/BCL-2 ratio (Figure 6C,D). JC-1 staining further showed that rapamycin alleviated the loss of mitochondrial membrane potential (ΔΨm) induced by REDD1 silencing (Figure 6E,F), while flow cytometry demonstrated a reduced apoptotic fraction (Figure 6G,H). Together, REDD1 modulated apoptosis of aortic vascular smooth muscle cells via mTOR regulation. In parallel, changes in mitochondrial membrane potential and autophagy-related indicators were observed, which may represent the downstream sequelae of REDD1/mTOR signaling.

## 4. Discussion

Loss of medial vascular smooth muscle cells (VSMCs) is a cardinal feature of aortic dissection (AD), reducing the cellular content of the media and disrupting extracellular matrix architecture [3]. Under persistent mechanical stress, inflammation, oxidative stress, and other insults, VSMCs become dysfunctional, and a subset subsequently undergoes irreversible forms of cell death, including apoptosis, pyroptosis, and ferroptosis [30,31,32]. As a fundamental mode of VSMC loss, apoptosis is particularly relevant to AD pathogenesis because its signaling machinery serves as a convergence point for multiple upstream pathological stimuli [33]. REDD1 was originally identified by Ellisen et al. [13] as a transcriptional target of p53 and was subsequently classified as a member of the REDD family. Its expression is strongly induced by diverse cellular stressors, including hypoxia, DNA damage, energy stress, and glucocorticoid exposure. REDD1 coordinates metabolic adaptation and cell-survival signaling under stress, although its effects are highly context dependent. In cancer-related studies, Mora-Molina et al. [34] found that REDD1 protected against endoplasmic-reticulum-stress-induced apoptosis by inhibiting mTORC1 and regulating tumor necrosis factor-related apoptosis-inducing ligand receptor 2/death receptor 5 (TRAILR2/DR5) expression. By contrast, Fang et al. [35] reported that REDD1 knockdown in lung adenocarcinoma cells reversed hypoxia-induced suppression of apoptosis and restored apoptosis. In diabetes-related research, Miller et al. [36] demonstrated that REDD1 deficiency prevented hyperglycemia-induced retinal neuronal apoptosis and visual dysfunction. Consistent with these observations, our single-cell analysis showed a significant association between REDD1 expression and VSMC apoptosis in patients with AD, suggesting that REDD1 may influence AD progression by regulating VSMC apoptosis [37]. In the present study, REDD1 expression was markedly upregulated in aortic tissues from patients with AD and from mice with beta-aminopropionitrile (BAPN)-induced AD, with REDD1 predominantly localized to medial VSMCs. This upregulation was accompanied by activation of apoptotic signaling. REDD1 silencing in BAPN-fed mice markedly exacerbated the AD phenotype, as evidenced by greater aortic dilatation, larger intramural hematomas, reduced survival, and increased VSMC loss; these changes were accompanied by further activation of apoptotic signaling. In cultured VSMCs, REDD1 knockdown likewise aggravated apoptosis and was associated with a reduction in mitochondrial membrane potential, suggesting that REDD1 may exert a cytoprotective effect by preserving mitochondrial membrane potential. Collectively, these findings suggest that REDD1 is more likely to exert a homeostatic protective role rather than a pro-apoptotic effect in aortic dissection, which provides an initial characterization of REDD1’s function in this aortic pathology.

Multiple studies of aortic disease have linked dysregulated autophagy to deterioration of the VSMC phenotype and instability of the aortic wall [8]. In a cell-specific genetic study, Clément et al. [38] showed that autophagy-related5 (ATG5) deficiency was associated with reduced LC3B, increased p62, a greater number of active caspase-3-positive medial VSMCs, impaired cell survival and proliferation, and heightened endoplasmic reticulum stress and IRE1α-dependent inflammation, ultimately leading to more severe aortic lesions. Pu et al. [39] reported that MIF suppressed autophagic flux through activation of AKT/mTOR signaling and promoted a synthetic VSMC phenotype, whereas rapamycin reversed MIF-induced phenotypic switching and mitigated MIF-driven AD pathology. Although these findings support a cytoprotective role for intact basal autophagic flux [40], the causal relationship between autophagy and VSMC apoptosis remains poorly defined. In response to pathological stimuli, autophagy may act upstream of apoptosis or operate in parallel with it [10,40,41,42]. In the present study, activation of apoptotic signaling in both human and murine AD tissues coincided with changes in core static autophagy-related markers. Specifically, increased LC3B and decreased p62 constituted a static marker profile suggestive of enhanced autophagic activity in the dissection model [43]. In AD mice and cultured VSMCs, REDD1 silencing further enhanced apoptotic signaling and produced a static autophagy-related marker profile indicative of reduced autophagic activity, characterized by decreased LC3B and increased p62. We therefore speculate that REDD1 silencing may disrupt basal autophagic levels and thereby exacerbate AD progression.

The mechanistic relationship between mTOR and autophagy has been extensively characterized [44]. Kim et al. [45] demonstrated in cellular and biochemical experiments that, under nutrient-replete conditions, mTORC1 directly phosphorylates ULK1 at Ser757 and weakens the interaction between AMPK and ULK1. During glucose deprivation, AMPK activates ULK1 through phosphorylation at sites including Ser317 and Ser777, thereby promoting autophagy initiation. Roczniak-Ferguson et al. [46] further showed that mTORC1 inhibition promotes the nuclear translocation of TFEB and induces the expression of lysosomal and autophagy-related genes, resulting in an expansion of cellular degradative capacity. Aberrant mTOR phosphorylation has also been reported in AD, and mTOR activation has been implicated in vascular wall remodeling [29]. Nevertheless, the upstream signals that drive aberrant mTOR activation, as well as whether its downstream effects are mediated through autophagy regulation or direct modulation of apoptotic pathways, remain controversial. An alternative view attributes autophagic dysfunction in AD to defective lysosomal acidification rather than abnormalities in upstream signaling [47]. These discrepancies underscore the need to define the relevant upstream regulatory mechanisms in AD. Functionally, REDD1 is a well-established negative regulator of mTORC1 signaling. By inhibiting protein kinase B (AKT) phosphorylation, REDD1 relieves AKT-mediated inhibition of tuberous sclerosis complex 2 (TSC2), thereby enhancing the GTPase-activating activity of the tuberous sclerosis complex 1/2 (TSC1/TSC2) and ultimately suppressing Rheb-dependent mTORC1 activation [18,44]. However, whether mTOR is regulated by REDD1 in AD has remained unknown. In cultured VSMCs, REDD1 silencing aggravated apoptosis in parallel with a marked increase in mTOR phosphorylation and produced a static autophagy-related marker profile indicative of reduced autophagic activity, characterized by decreased LC3B and increased p62. This profile was consistent with established effects of mTOR activation on autophagy. Following treatment with rapamycin (RAPA), apoptotic signaling was attenuated, mitochondrial membrane potential was partially restored, and the changes in static autophagy-related markers were reversed. These findings indicate that, in VSMCs, REDD1 modulates apoptosis by regulating mTOR phosphorylation. The reciprocal shifts in static autophagy-related markers further suggest that REDD1 may influence autophagy-related activity through mTOR signaling and thereby affect VSMC fate. Together, these results provide a new perspective on the upstream regulation of VSMC apoptosis in AD.

Several limitations of this study should be acknowledged. In the animal experiments, the systemic AAV-shRNA-mediated gene silencing strategy precluded exclusion of contributions from other aortic wall cell types, including fibroblasts and endothelial cells [48,49,50]. REDD1 knockdown has also been reported to attenuate oxidative injury in vascular endothelial cells and to reduce the production of multiple proinflammatory factors and adhesion molecules [51]. Such effects in nontarget cell types should therefore be considered when interpreting the phenotype associated with REDD1 silencing. In addition, autophagy plays a complex and context-dependent role in the progression of aortic disease, and we were unable to establish a definitive causal relationship between autophagy and apoptosis in VSMCs. In future studies, we will use an adenoviral vector targeting aortic medial smooth muscle cells to silence REDD1 and will investigate the REDD1/mTOR signaling axis identified in this study using more rigorous and definitive mechanistic approaches.

In conclusion, this study expands the functional scope of REDD1 by identifying it as a key regulator of aortic medial homeostasis rather than merely a conventional stress-response factor. Our findings demonstrate that REDD1 silencing promotes the development and progression of AD in BAPN-fed mice, while the in vitro data indicate that REDD1 modulates mitochondrial membrane potential and VSMC apoptosis through regulation of mTOR phosphorylation. Changes in core static autophagy-related indices further suggest that REDD1 may influence AD progression by modulating autophagy-related activity. Collectively, these findings deepen our understanding of AD pathogenesis and provide a mechanistic rationale for therapeutic strategies targeting the REDD1/mTOR signaling axis.

## Data Availability

The datasets used and/or analyzed during this study are available from the corresponding author on reasonable request. The dataset supporting the conclusions of this article is available in the Gene Expression Omnibus repository under accession number GSE222318 (https://www.ncbi.nlm.nih.gov/geo/query/acc.cgi?acc=GSE222318, accessed on 7 July 2025).

## Supplementary Materials

The following supporting information can be downloaded at https://www.mdpi.com/article/10.3390/biomedicines14092061/s1, Table S1: Gene Set for Analysis of the Relationship Between Autophagy and Apoptosis.

## Abbreviations

The following abbreviations are used in this manuscript:

AAV	Adeno-associated virus
AD	Aortic dissection
AKT	Protein kinase B
Ang II	Angiotensin II
ANOVA	Analysis of variance
ATCC	American Type Culture Collection
ATG5	Autophagy-related5
BAPN	β-Aminopropionitrile
BAX	BCL-2-associated X protein
BCA	Bicinchoninic acid
BCL-2	B-cell lymphoma 2
DAPI	4′,6-Diamidino-2-phenylindole
DDIT4	DNA damage-inducible transcript 4
DMEM	Dulbecco’s modified Eagle’s medium
DMSO	Dimethyl sulfoxide
ECL	Enhanced chemiluminescence
EVG	Elastica van Gieson
FITC	Fluorescein isothiocyanate
H&E	Hematoxylin and eosin
HRP	Horseradish peroxidase
JC-1	5,5′,6,6′-Tetrachloro-1,1′,3,3′-tetraethylbenzimidazolylcarbocyanine iodide
LC3B	Microtubule-associated protein 1 light chain 3 beta
MIF	Migration Inhibitory Factor
MOVAS	Mouse aortic smooth muscle cells
mTOR	Mechanistic target of rapamycin
mTORC1	Mechanistic target of rapamycin complex 1
NIH	National Institutes of Health
p62	Sequestosome 1
PBS	Phosphate-buffered saline
PCA	Principal component analysis
PI	Propidium iodide
PMSF	Phenylmethylsulfonyl fluoride
p-mTOR	Phosphorylated mechanistic target of rapamycin
PVDF	Polyvinylidene difluoride
RAPA	Rapamycin
REDD1	Regulated in development and DNA damage responses 1
Rheb	Ras homolog enriched in brain
RIPA	Radioimmunoprecipitation assay
SD	Standard deviation
SDS	Sodium dodecyl sulfate
SDS–PAGE	Sodium dodecyl sulfate–polyacrylamide gel electrophoresis
shRNA	Short hairpin RNA
SPF	Specific pathogen-free
TSC1	Tuberous sclerosis complex 1
TSC2	Tuberous sclerosis complex 2
UMAP	Uniform manifold approximation and projection
VSMCs	Vascular smooth muscle cells
α-SMA	α-Smooth muscle actin
ΔΨm	Mitochondrial membrane potential
